# Supplementary material for: The role of community-level men’s and women’s inequitable gender norms on women’s empowerment in India: A multilevel analysis using India’s National Family Health Survey–5
Source: PLoS One. 2024 Dec 11;19(12):e0312465. doi: 10.1371/journal.pone.0312465 (PMC11633985; doi:10.1371/journal.pone.0312465)
Supplement: Attachment — Submitted filename: Reviewer file_Aug14.pdf [file pone.0312465.s001.pdf]

The role of community-level men's and women's inequitable gender norms on women's empowerment in India: A multilevel analysis using India's National Family Health Survey–5

Reviewer file

Abstract	
Background: Lower empowerment of women is a human rights issue with adverse public health implications.	Though the concept of human rights in invoked in the abstract, in the main ms. at no point is this brought up. Human rights is too serious and important an issue and a concept to be invoked casually. Strongly suggest using only concepts that are used in the ms. to be used in the abstract.
	Would be very useful to have a few words to 'locate' the concept of gender norms in the Background, prior to invoking in in the Objective.
Introduction	
Gender systems often tend to be patriarchal, ascribing greater value to masculinity rather than femininity	Femininity is ascribed "greater value" on occasions such as child bearing or upholding the honour of the family. So, it is the context or the issue that determines if masculinity or femininity is ascribed greater value.
nowhere else is this more relevant than in India.	I would certainly say, it is way more relevant in Afghanistan or Palestine at the moment!<br>Well, my "reaction" aside, I suggest that we avoid such opinions that are not backed by solid evidence. If author(s) decide to use this line, they would then need to explain WHY is this most relevant for India. The "rational" provided at the moment convinces us why India should be studied, but not why this is most relevant for India among 192 countries.
The most apparent evidence of this is India's unevenly skewed sex ratios at birth<br>(favoring boys) and the persistent favoritism towards boys displayed in care-seeking practices,	Would be good to mention the sex ratio at birth, the latest data on that. while it might be there in the two references, it is better to embed it in the text itself.
In patriarchal societies such as India, we hypothesize that community-level gender norms are a byproduct of men's beliefs about women's roles and duties — especially in the context of cultural values, traditional family roles, gender values, and gender order.	The basis of this hypothesis is not clear.<br>Evidence (from DHS in many countries, including India's NFHS) shows that the % of women justifying wife beating is higher than % men justifying it. This, and a whole lot of other evidence shows that it is not just men who have harmful beliefs about women's roles and duties; women themselves are convinced about their roles as home makers or the need to listen to the husband or take his permission for any major decision. So, I am not sure what is this hypothesis informed by.
Since women also live in the same communities as men, we hypothesized that women's attitudes towards	Would be good to "club" all the hypothesis at one place, at the end of the Introduction.

gender equality might also add to the normative environment, which could influence women's empowerment. We hypothesized that women residing in communities with higher inequitable men's gender norms and inequitable women's gender norms (modeled independently) will be more likely to have lower freedom of movement, lesser decision-making power, lower economic resources, and lower health empowerment.	
Discussion	
This underscores the significant influence of normative environment on women's empowerment, irrespective of whether men or women uphold patriarchal norms and regressive attitudes.	This sentence needs unpacking
	Discussion section should speak more to the global literature -
This could plausibly be because even though women may be economically empowered with access to economic resources, inequitable gender norms could dictate the economic roles women should play	Can the authors discuss a bit more on what they mean by "dictate the economic roles women should play"? what economic roles are women, even those with access to economic resources, expected to play?
In contexts where patriarchal systems are strong, such as India, analysis of community norms and their effect on various dimensions of women's empowerment provides important evidence of the need to address women's empowerment not only at the individual level but also at the community level.	Can authors suggest some approaches for community level analysis of the various dimensions of women's empowerment? We keep leaving all the difficult parts to "future research" and nobody gets around to that piece! So, if we can also suggest a few approaches or put forth some recommendations that are practical and doable, that would be a strong contribution to literature.

Our findings align consistently with research from other low-and-middle-income countries,	While authors say findings are consistent with evidence from other LMICs, the examples discussed are from 2-3 countries. Could we have some more discussion please?
….. highlighting the role of intergenerational transmission in the formation of gender attitudes.	Could the authors substantiate the argument about intergenerational transmission of gender attitudes a bit more?
One study in India<br>addressed social and gender norms to reduce women's anemia rates….	Do we know what the intervention found? A line, reflecting on the evidence generated by this study, would be very helpful.
we aimed to understand how the different sexes imbibe gender attitudes and impact women's empowerment differently	I am not convinced that the paper understands **how** the different sexes imbibe gender attitudes … the paper only studies the associations, not any in-depth analysis of the 'how' different genders imbibe gender attitudes … please edit this line.
Consolidating women's empowerment into a single-scale measure presents several limitations, such as potential measurement<br>errors and the risk of overlooking the multifaceted nature of the concept [	This is a "limitation" and should be part of the paragraph that talks of the Limitations.
